# Functional 2D MXene Inks for Wearable Electronics

**DOI:** 10.3390/ma14216603

**Published:** 2021-11-02

**Authors:** Bouchaib Zazoum, Abdel Bachri, Jamal Nayfeh

**Affiliations:** 1 Department of Mechanical Engineering, Prince Mohammad Bin Fahd University, Al Khobar 31952, Saudi Arabia; jnayfeh@pmu.edu.sa; 2 Department of Physics & Engineering, Southern Arkansas University, Magnolia, AR 71753, USA; agbachri@saumag.edu

**Keywords:** inks printing, 2D MXene, wearable electronics, inks formulation, MSC

## Abstract

Inks printing is an innovative and practicable technology capable of fabricating the next generation of flexible functional systems with various designs and desired architectures. As a result, inks printing is extremely attractive in the development of printed wearables, including wearable sensors, micro supercapacitor (MSC) electrodes, electromagnetic shielding, and thin-film batteries. The discovery of Ti_3_C_2_T_x_ in 2011, a 2D material known as a MXene, which is a compound composed of layered nitrides, carbides, or carbonitrides of transition metals, has attracted significant interest within the research community because of its exceptional physical and chemical properties. MXene has high metallic conductivity of transition metal carbides combined with hydrophilic behavior due to its surface terminated functional groups, all of which make it an excellent candidate for promising inks printing applications. This paper reviews recent progress in the development of 2D MXene inks, including synthesis procedures, inks formulation and performance, and printing methods. Further, the review briefly provides an overview of future guidelines for the study of this new generation of 2D materials.

## 1. Introduction

The utilization of 2D nanomaterials has attracted increasing attention from the materials science and engineering research communities owing to their exceptional chemical, electrical, thermal, and optical behaviors, which make these materials useful for next generation applications.

Ink printing of 2D materials offers promising manufacturing technologies in many applications, including wearable electronics, micro supercapacitor (MSC) electrodes, thin-film batteries, flexible electronics, electronic skins, health monitoring sensors for implantable medical devices, and microrobots [1,2,3,4,5,6,7]. Compared to traditional manufacturing methods, ink printing techniques include inkjet printing, screen printing, and extrusion printing or stamping, all of which are fast, low cost, and easy processes that permit high printing resolution and flexible digital patterning. A further advantage in using ink printing is the integration of functional inks with appropriate properties, especially in terms of viscosity, density, and surface tension, all of which are critical for jettability [8,9,10,11,12]. Because of this, current printing technologies should be enhanced and new design approaches established [13].

Screen printing has weak overlay precision, which can adversely affect the uniformity of the printed pattern [10,14,15,16,17,18]. Inkjet printing, by contrast, is an extensively utilized method that provides higher printing resolution. Inkjet printing is digital, and therefore is more accurate in deposing materials patterning. Since it is controlled by computer, inkjet printing could easily reduce waste generation.

Nevertheless, in terms of mass production, inkjet printing is still used far less than other conventional methods [14,19]. One reason for this may be that the nature and content of inks can easily block printing nozzles, which could weaken the fabrication productivity. Substantial effort, therefore, must be focused on ink formulation, including filler functionalization and thermal and rheological properties, to develop and design appropriate inks consistent with the target patterning form and type of substrates.

**Figure 1:**
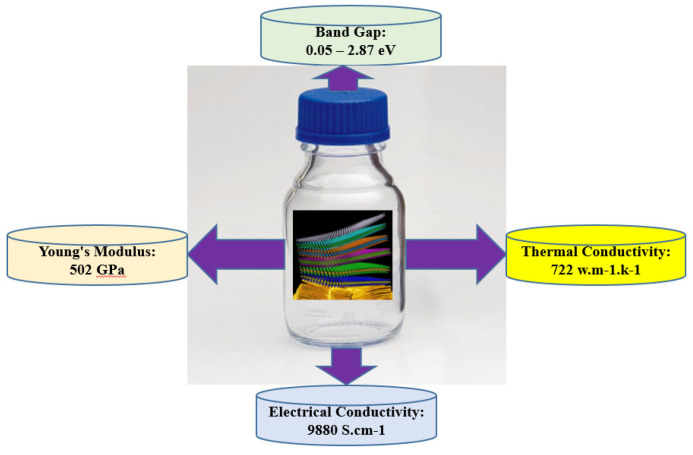
Summary of the mechanical, electronic, thermal, and electrical properties of MXenes.

Since their discovery in 2011 at Drexel University [20], researchers have explored 2D transition metal carbides and nitrides (MXene), materials that have high intrinsic thermal and electrical conductivity, as well as excellent hydrophilic and mechanical properties (Figure 1). This research has encompassed many applications, including SMC electrodes, sensors, and electronic devices, as well as in the areas of aerospace and medicine. The 2D MXene is synthesized using etching methods by removing the A-group element layers from the precursor’s ternary carbides and nitrides, or MAX phases (where M denotes a transition metal and X represents carbon and/or nitrogen).

The chemical formula of MXene materials can be written as Mn+1XnTx, where n = 1, 2, or 3, M is the transition metal (Ti, Zr, Hf, V, Nb, Ta, Cr, etc.), X represents carbon C or nitrogen N, and Tx is the surface terminated functional group, such as hydroxyl (-OH), oxygen (-O) or fluorine (-F) [21], which render these materials hydrophilic, permitting them to be well dispersed into aqueous inks. To date, there are numerous MXenes that have been prepared [22,23,24], although most previous work on inks printing has studied Ti_3_C_2_, because of its physical and chemical properties [25,26,27,28,29,30,31,32].

Despite the growing interest in the MXene inks, there is a lack of comprehensive reviews covering the synthesis procedures, formulation, performances, and printing methods of these inks. This paper presents a summary of the current progress in developing 2D MXene inks for printing applications. After a short introduction to synthesis procedures, we will discuss the effect of etching methods and solvent selection on the final structure of MXene inks. We will then describe their pertinent rheological, thermal, and electronic properties, and their usefulness in the most promising printing methods, such as screen printing, inkjet printing, stamping, and 3D printing. Finally, we will briefly provide an outlook on future guidelines for studying the next generations of advanced 2D materials.

## 2. MXene Synthesis

### 2.1. Etching Procedures

**Figure 2:**
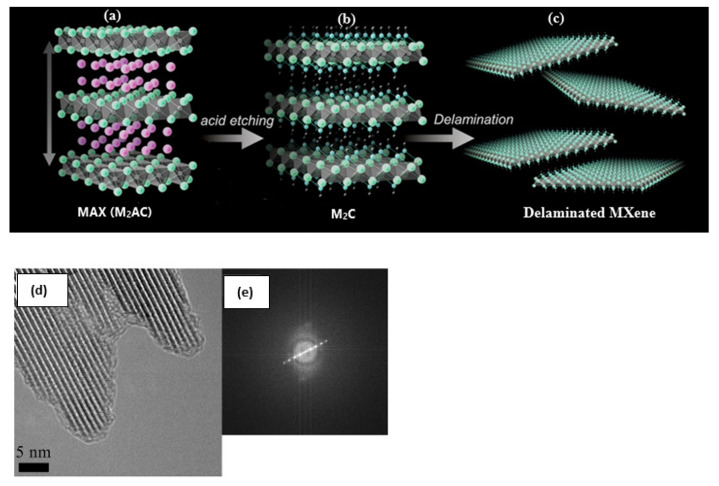
Schematic illustration showing (**a**–**c**) synthesis of delaminated MXene. Modified from [25], with permission from Wiley-VCH. 2016. (**d**) High Resolution TEM images with (**e**) the Fourier Transform, for delaminated Ti_3_C_2_ Mxene [28]. Copyright (2017), with permission from Elsevier.

Synthesis of MXene initiates with wet chemical etching using hydrofluoric acid (HF), bifluoride-based etchants (NH_4_HF_2_), or HF containing salt to remove the A layers from ternary carbides and nitrides (Figure 2a,b) [25] which have the chemical formula of M_n+1_AX_n_ (n = 1–3) or MAX phases, where M is a transition metal (Ti, Zr, Hf, V, Nb, Cr, etc.), A belongs to an A-group element and X represents C and/or N. This process is possible since the metallic bond between the two elements M and A is more reactive compared to the bond between M and X [24]. B. Anasori et al. [21] reported that an increase in M necessitates a strong etchant with longer etching time. In the case of Ti_3_AlC_2_, the HF etching process could be summarized by the following equations:

(1) $${\mathrm{Ti}}_{3}{\mathrm{AlC}}_{2}\;\left(s\right)+3\mathrm{HF}\;\left(\mathrm{aq}\right)\to {\mathrm{Ti}}_{3}C_{2\;}\left(s\right)+{\mathrm{AlF}}_{3}\left(\mathrm{aq}\right)+\frac{3}{2}H_{2}\;\left(g\right)$$

(2) $${\mathrm{Ti}}_{3}C_{2}\;\left(s\right)+2\mathrm{HF}\;\left(\mathrm{aq}\right)\to {\mathrm{Ti}}_{3}C_{2}F_{2}\;\left(s\right)+H_{2}\;\left(g\right)$$

(3) $${\mathrm{Ti}}_{3}C_{2}\;\left(s\right)+2H_{2}O\left(\mathrm{aq}\right)\to {\mathrm{Ti}}_{3}C_{2}\left(\mathrm{OH}\right)\left(s\right)+H_{2}\left(g\right)$$

The mild etchant NH_4_HF_2_ is also employed to extract the Al layer from Ti_3_AlC_2_; however, in this method, one needs a longer time and higher temperatures to dry the water that was introduced between the Ti_3_C_2_T_x_ layers during the etching process [21]. These reactions are described as follow [26,27]:

(4) $${\mathrm{Ti}}_{3}{\mathrm{AlC}}_{2}\;\left(s\right)+3{\mathrm{NH}}_{4}{\mathrm{HF}}_{2}\left(\mathrm{aq}\right)\to {\mathrm{Ti}}_{3}C_{2}\;\left(s\right)+{\left({\mathrm{NH}}_{4}\right)}_{3}{\mathrm{AlF}}_{6}\left(\mathrm{aq}\right)+\frac{3}{2}H_{2}\left(g\right)$$

(5) $${\mathrm{Ti}}_{3}C_{2}\;\left(s\right)+{\mathrm{aNH}}_{4}{\mathrm{HF}}_{2}\left(\mathrm{aq}\right)+{\mathrm{bH}}_{2}O\left(\mathrm{aq}\right)\to {\left({\mathrm{NH}}_{3}\right)}_{c}({\left({\mathrm{NH}}_{4}\right)}_{d}{\mathrm{Ti}}_{3}C_{2}{\left(\mathrm{OH}\right)}_{x}F_{y}\left(s\right)$$

### 2.2. Intercalation and Delamination

The intercalation and delamination process requires an appropriate solvent for the material and intercalant. First, a selective intercalant such as dimethyl sulfoxide (DMSO), tetrabutylammonium hydroxide (TBAOH), or tetramethylammonium hydroxide (TMAOH) is introduced to increase the spacing distance between the layers of Ti_3_C_2_T_x,_ followed by a sonication process to delaminate multilayered Ti_3_C_2_T_x_ into separate 2D single-flakes [25,28] (Figure 2c–e).

## 3. Formulation of MXene Inks

### 3.1. Selection of Etching Method

**Figure 3:**
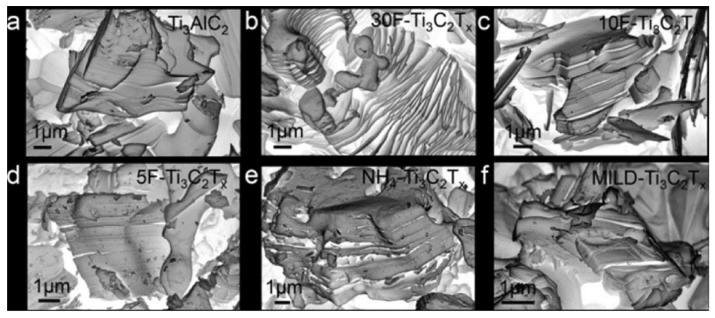
SEM micrographs show (**a**) Ti_3_AlC_2_ (MAX) powder and MAX powder etched with (**b**) 30 wt.% HF; (**c**) 10 wt.% HF and (**d**) 5 wt.% HF; (**e**) ammonium hydrogen fluoride; and (**f**) mild LiF/HCl. Modified from [29], with permission from American Chemical Society, 2017.

The degree of exfoliation or intercalation of the MXene layers can be determined by transmission electron microscopy (TEM), scanning electron microscope (SEM), or X-Ray diffraction. Alhabeb et al. [29] studied the effect of etchant choice and etchant concentration on the final microstructure of MXene. Figure 3 shows SEM micrographs for (a) the MAX phase; (b) the MAX phase etched with 30 wt.% HF; (c) with 10 wt.% HF; and (d) with 5 wt.% HF. The study finds that in the absence of etching (Figure 3a), the existence of the MAX phase aggregates can be observed at the micrometer level, and consequently, the structure is poorly intercalated. However, when the MAX phase was etched with HF, that interlayer spacing of MXene expanded as the concentration of HF increased (Figure 3b–d). These results also show that in the case of ammonium hydrogen fluoride and mild LiF/HCl etching methods, the opening of MXene layers was negligible, as shown in Figure 3e,f. In order to confirm the microscopic results, the morphology of the materials was examined by X-ray diffraction.

**Figure 4:**
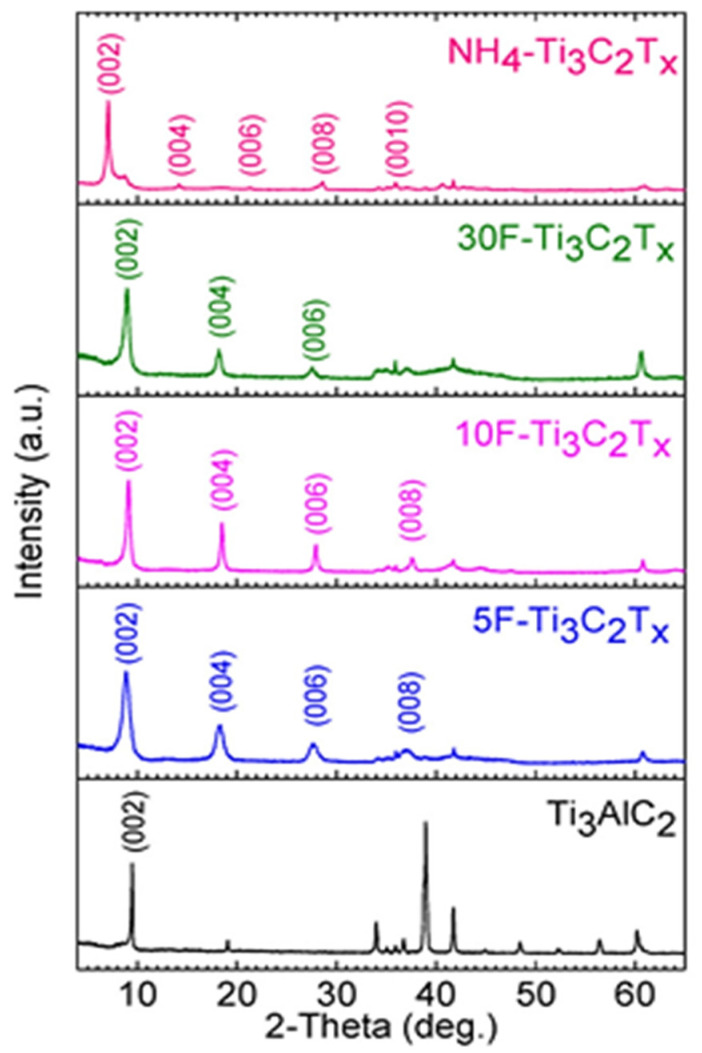
X-ray diffraction pattern of Ti_3_AlC_2_ (MAX) powder and MAX powder etched with 30, 10, and 5 wt.% HF and ammonium hydrogen fluoride etching routes [29]. Copyright 2017, American Chemical Society.

Figure 4 shows the X-ray diffraction patterns of (a) the MAX phase; (b), the MAX phase etched with 30 wt.% HF; (c) with 10 wt.% HF; (d) with 5 wt.% HF; and (e) ammonium hydrogen fluoride. The results show clearly that when the MAX phase is etched with HF, the residual peaks of Ti_3_AlC_2_ are not detected and the diffraction peak corresponding to basal plane (002) is shifted to a smaller angle, indicating an increase in interlayer spacing (d) of the galleries of MXene. In the HF etching process, results show that the d-spacing expansion appears to be related to HF concentration, with d-spacing of MXene layers increasing with higher levels of HF concentration. On the other hand, and contrarily to SEM observation, the XRD results show that the ammonium hydrogen fluoride etching method leads to the high d spacing. This is due to the intercalation of the cations NH^4+^ during the etching process. However, the NH^4+^ could introduce water molecules into the MXene layers, which could complicate the drying of the resulting MXene [29].

### 3.2. Solvent Selection

**Figure 5:**
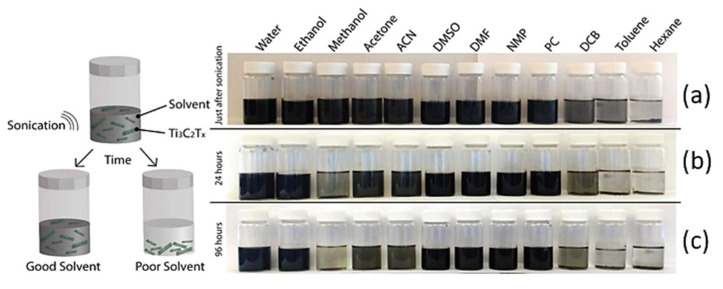
Monitoring the dispersion stability of 50% HF-etched Ti_3_C_2_T_x_ in 12 different solvents: (**a**) just after sonification process, (**b**) 24 h after sonication, and (**c**) 96 h after sonication [30]. Copyright 2017 American Chemical Society.

Usually, MXene inks can be produced in the form of powder or a colloidal solution of the MXene layer dispersed in appropriate solvent. MXene inks are known to have a good degree of dispersion in water or in various organic solvents. However, it was reported by Maleski et al. [30] that storing MXene in organic solvents allows the dispersion of MXene to remain stable for longer period. The same authors studied the effect of solvent medium on the stability of MAX etched by 50 wt.% HF in 12 different solvents, including deionized water, polar solvents (ethanol (EtOH), methanol (MeOH), acetone (ACE), acetonitrile (ACN), N,N-dimethylformamide (DMF), dimethyl sulfoxide (DMSO), N-methyl-2-pyrrolidone (NMP), propylene carbonate (PC)), less polar to nonpolar solvents (hexane (HEX), toluene (TOL), and 1,2-dichlorobenzene (DCB)) (Figure 5). The authors visually evaluated the quality of dispersion of MXene in the different solvents. The results clearly show that after sonication process, a good dispersion was noted in all polar solvents; however, bad quality dispersion was achieved in the other three less polar and nonpolar solvents. After 24 h of sonication, the quality of dispersion for methanol dramatically decreased. Similarly, a degraded dispersion of MXene in the solvents acetone and ACN was observed after 96 h. Further, high dispersion stability was observed even after 96 h for the solvents deionized water, ethanol, DMSO, DMF, NMP, and PC.

**Figure 6:**
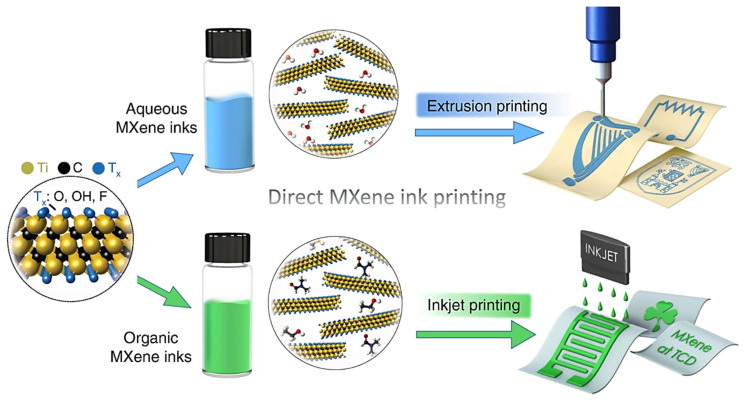
Schematic description of MXene ink printing. (**Top**) The Ti_3_C_2_T*_x_* aqueous ink (Ti_3_C_2_T_x_ dispersed in water) is appropriate for extrusion printing. (**Bottom**) The Ti_3_C_2_T*_x_* organic ink (Ti_3_C_2_T*_x_* dispersed in ethanol) is suitable for inkjet printing. [31]. Copyright 2019, Springer Nature.

In term of application, the formulation of MXene inks is critical. The investigation of Zhang et al. [31] showed that deionized water MXene inks are suitable for the extrusion printing technique, while organic MXene inks are designed for the inkjet printing process (Figure 6). Optimization of surface tension is also critical to achieve printable inks. Inkjet printing requires optimized surface tension to avoid any spontaneous dripping or the ejection of droplets.

## 4. Performances of MXene Inks

### 4.1. Rheological Behaviors of MXene Inks

**Figure 7:**
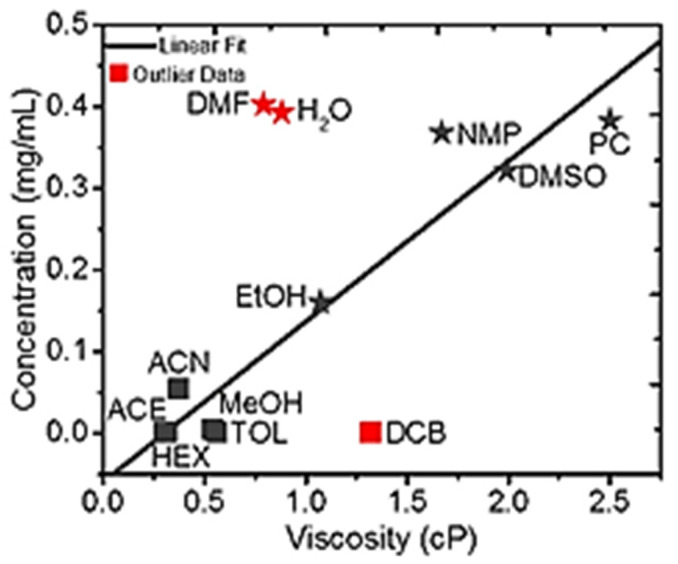
Linear relationship between MXene concentration and solvent viscosity for all solvents excepting DCB, DMF, and water [30]. Copyright 2017 American Chemical Society.

Understanding the rheological behaviors of the MXene solution is an important factor in developing MXene inks intended for specific applications of the manufacturing process. The viscosity of the solvent plays a major role in determining the quality and the ease of the inkjet printing, extrusion, or coating printing process. The viscoelastic properties of MXene dispersed in various solvents has already been studied (Figure 7) [30]. The results of that study showed a linear relationship between MXene concentration and solvent viscosity for the solvents EtOH, MeOH, ACE, ACN, DMSO, NMP, PC, HEX, and TOL; however, for the solvents water, DMF, and DCB, this linear relationship was found to be poor. Consequently, it is very important to take into consideration solvent viscosity when preparing the appropriate inks for various fabrication techniques.

**Figure 8:**
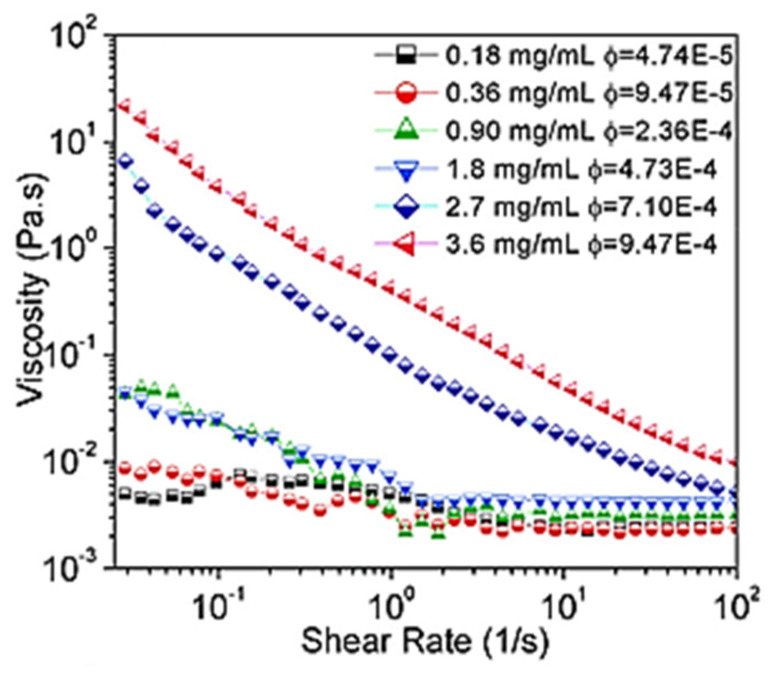
Variation of the viscosity of single-layer MXene flake as function of shear rate and with concentration ranging from 0.18 to 3.6 mg/mL [32]. Copyright 2018 American Chemical Society.

Akusum et al. [32] studied the rheology of multilayer MXene (Ti_3_C_2_T_x_) dissolved in deionized water. Figure 8 displays the variation of the viscosity of single-layer MXene (Ti_3_C_2_T_x_) as a function of shear rate at different MXene (Ti_3_C_2_T_x_) concentration levels (mg/mL) (or volume fraction (ϕ)). The figure indicates that the viscosity remains constant over the entire frequency for those solutions with low concentrations of deionized water (0.18 and 0.36); however, the level of viscosity decreased with shear rate for those solutions with a concentration higher than 0.36. This behavior is expected for colloidal solutions [33]. The results further indicated that over the whole shear rate domain, the viscosity of the MXene increased with increasing MXene concentration. This is mostly related to viscosity of MXene comparing to that of deionized water.

### 4.2. Thermal Stability of MXene Inks

**Figure 9:**
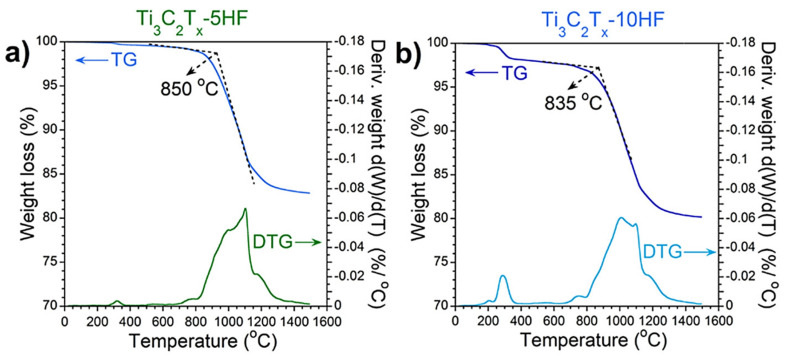

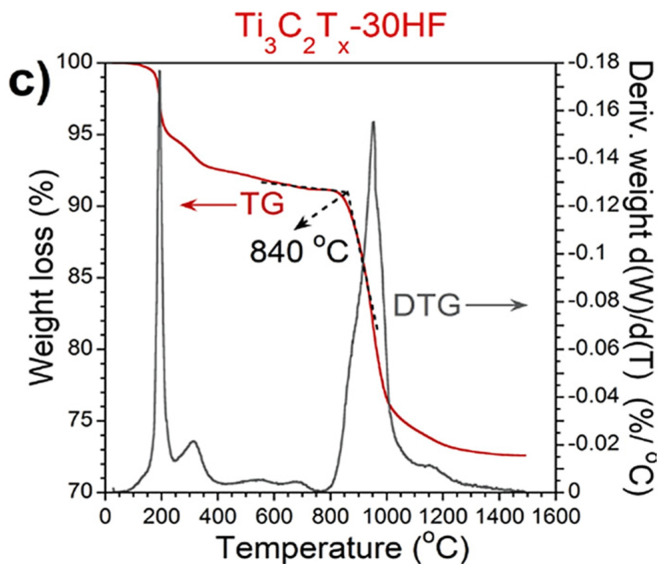
Thermal gravimetric (TG) plots with derivatives (DTG) for MAX powder etched with (**a**) 5 wt.% HF, (**b**) 10 wt.% HF, and (**c**) 30 wt.% HF [34]. Copyright 2019, American Chemical Society.

In various applications and manufacturing processes, MXene can be exposed to a large range of temperatures, which influence its thermal properties. Seredych et al. [34] investigated the thermal behavior of MXene using thermal gravimetric analysis with mass spectrometry (TA−MS), which is an established technique for studying the residue weight of materials under various temperatures. Figure 9 shows TA−MS curves of Ti_3_C_2_T_x_ multilayer MXene etched with (a) 5 wt.% HF, (b) 10 wt.% HF, and (c) 30 wt.% HF. As the figure indicates, in all three concentrations an entrapped structural H_2_O peak was detected at 320 °C. As the HF concertation increased, a new peak appeared at 200 °C.

Further, the onset temperature related to water release was shifted from 320 °C to lower temperatures (100 °C). This is mostly due to the water introduced into multilayer Ti_3_C_2_T_x_ at high HF concentrations. The content of this water was found to increase with higher levels of HF concentration. This can be explained by the fact that d spacing between MXene layers expanded with HF concertation. The results also show that for all HF concentration levels, the structure of Ti_3_C_2_T_x_ MXene remains stable up to 800 °C, but above this temperature, the sample begins to degrade.

### 4.3. Electronic Properties

The electronic performance of MXene is important when the Mxene materials are employed in applications involving wearable electronics devices. It is well known that the conductivity of MXenes depends mostly on their composition. In general, all MXene materials are metallic, whereas after surface modification some of them become semiconductors [35,36]. For example, the Nb_2_C, Nb_2_CF_2_, Nb_2_C(OH)_2_, and Ti_3_C_2_ are classified as metallic conductors, though Ti_3_C_2_T_x_ is considered as semiconductor material. The electronic band structures of M_n+1_X_n_ are dense, h resulting in the metallic conductivity of several M_n+1_Xn structures. The narrow band gap of the Ti_3_C_2_T_x_ structure shows that the structure is semiconductive [36]. Further, the thickness of the Ti_3_C_2_T_x_ structure has a significant effect on the value of resistance and transmittance; consequently, the optoelectronic properties are important. It has been shown that the electrical conductivity of the Ti_3_C_2_T_x_ can reach a value of 6500 S cm^−1^ [37,38].

Rajan et al. studied the electronic properties of 2D Mxene by estimating the band gaps using statistical learning. In this study three regression algorithms including kernel ridge (KRR), support vector (SVR), Gaussian process (GPR), and bootstrap aggregating were used. The input parameters of the models were boiling and melting points, atomic radii, phases as well as bond lengths. Their results revealed that the GPR model predicts the band gap with lowest root-mean-squared error (RMSE) of 0.14 eV [39].

As discussed previously, Mxene materials are favorable candidate for electronic applications owing to their high electrical and thermal conductivity combined with hydrophilic properties. B. Lyu et al. have utilized Ti_3_C_2_T_x_ Mxene materials as contact electrodes in the organic thin film transistor (OTFT), with p-type and n-type channels [40]. The results of this work reported that the fabricated OFET using Ti_3_C_2_Tx Mxene 2D materials revealed exceptional device performance, including a max carrier mobility of almost 1 cm^2^/V·s and an on-off current ratio around 10^7^ for the p-type and n-type OFET.

Mxene 2D materials are also largely utilized as electrodes in supercapacitors owing to their 2D structure with vast specific surface area and high electrical conductivity. The electrodes made of MXene store charges mainly from the redox reaction [41]. Furthermore, these electrodes also display considerable capacitance at high scan rate. This explains how the specific surface area of 2D MXene has strong ability to store adequate electrostatic charges [42].

## 5. Printing Methods of MXene 2D Film

Depending on the applications, there are numerous methods for depositing structural patterned MXene film with controlled thickness on the substrate. To date, different printing and coating technologies have reportedly accomplished this objective [43,44,45,46]. This section summarizes the most widely used printing methods such as screen printing, inkjet printing, stamping, and 3D printing.

### 5.1. Screen Printing

Screen printing is employed for mass production of MSC, thin-film batteries, flexible electronics, and sensor electrodes due its stability and reproducibility [47]. In this technique, a stencil is placed between the ink and the substrate on which MXene inks patterns are to be deposed. The inks are pressed through a planar form stencil onto a desired substrate. The ink subsequently dries to form the desired patterns on the substrate. This process is repeated several times to realize multilayer deposition.

The resolution of these patterns is dependent on the resolution of the stencil through which the ink penetrates. It is preferable that inks are viscous and that they lose viscosity moderately when stress is applied to them. Li et al. [48] used hydrous ruthenium oxide, MXene nanosheet, and silver nanowire to develop a composite thixotropic ink to be used for screen printing. The researchers discovered that below the yield point, thixotropic ink and pure MXene ink both behave as solids. However, above the yield point, they act as liquids in reaction to shear stress. This behavior of MXene inks results in an easy flow of the inks through the stencil used for the screen -printing method. Silver nanowires (AgNWs) were found to be homogenous in the composite ink, improving the conductivity of the ink and preventing the sheets of MXene from restacking.

### 5.2. Inkjet Printing

Much faster than screen printing, inkjet printing is very commonly used as a noncontact and digital printing method for fabricating numerous devices, including MSC, sensors, and transistors [49,50,51,52]. However, there are several constraints associated with the inkjet printing method. A highly viscous ink is too difficult to eject, while ink with low viscosity causes too much splashing on the substrate surface, affecting the resolution and sharpness of the printed results.

The fluid parameter used to monitor drop formation is the Ohnesorge number (Oh) [53]. The inverse of this number is kept at a minimum value of 4 at the time of ejection; however, it should not exceed a specified threshold value to avoid splashing on the substrate surface [54]. That threshold value depends on the roughness and surface energy of the substrate. It was reported that the thickness of MXene ink has an effect on its rheological properties. Akuzum et al. [32] discovered that even at high levels of MXene content, multi-layer ink still showed considerable flowability. This behavior makes high concentration aqueous ink solutions suitable for techniques such as inkjet printing.

On the other hand, single-layer ink solutions exhibit elasticity at concentrations as low as 0.2 mg/mL. This behavior dictates that dilute solutions of single-layer MXene ink are suitable for spray or spin coating. A benefit of MXene inks is that they are stable without requiring any chemical additives, unlike ordinary inks that require surfactants. For the etching process to be followed in inkjet printing, water is used as a solvent for ink material. However, water has a high surface tension relative to most solvents. Methanol has a low surface tension, but MXene is not strongly dispersed in it, mainly because of its low polarity, resulting in a high value for the inverse of the Ohnesorge number, which is associated with poor droplet formation. This issue was first solved by Zhang et al. [31] who used AlO_x_-coated PET as substrates. Their study showed that for both high and low concentration MXene, inks exhibited the desirable resolution. The most interesting result of this study was that the ink was so adhesive to the substrate that it did not come off the substrate after scotch tape was applied and then removed from its surface. The printed MSC formed by inkjet printing has a capacitance of 562 F/cm^−3^ and an energy density of 0.32 µWh cm^−2^. These are the highest values achieved relative to other printed MSCs.

### 5.3. Stamping

Compared to other more conventional methods, stamping is considered one of the more promising and economical fabrication techniques for developing printed electronic devices. Hyun et al. [55] integrated stamping with inkjet printing. A self-aligning printing process was combined with a stamp for the fabrication of flexible MSC made of graphene. In the Self-aligned Capillary Assisted Lithography for Electronics (SCALE) process, the interdigitated pattern is printed on a PET film. This process is followed by ink printing graphene, which acts as the electrode material. This method can be used to produce the desired electrode directly. To fabricate the MSC, this electrode-active substance is transferred to the groove of the electrode pattern. The specific capacitance of the fabricated MSCs showed a high value of 268 μF/cm^2^ combined with exceptional stability under bending conditions.

Zhang et al. [56] have developed a stamping process for 2-D Ti_3_C_2_T_x_ MXene–based MSC. In their methodology, a 3D-printed stamp coated with MXene ink was printed on paper substrates and gel-electrolytes were cast. The resulting MSC displayed high capacitance values of 61 mF/cm^2^. This process is economical and produces different desired patterns with MXene ink and 3D-printed stamps.

### 5.4. 3D Printing Method

The method of 3D printing is considered as a form of Additive Manufacturing (AM). As the name suggests, it operates by adding material in a pattern to form a product, as opposed to subtractive manufacturing which operates by removing material. Obviously, since no material is removed in 3D printing, no material is wasted, making AM resource-conservative. Two primary modes for 3D printing are inks-based and light-based printing [57,58]. The light-based method, including stereolithography and laser sintering, showed high-resolution printing patterns. However, this technique has a limited printing speed. The types of material that are suitable for this method are also limited. Thermoplastic polymers resins are commonly employed for light-based 3D printing.

Ink-based 3D printing is implemented with a variety of methods, such as Fused Deposition Modeling (FDM), Direct-Ink-Writing (DIW) of soft materials, and binder jetting. These methods are suitable for a wide range of materials. FDM is the primary methodology incorporated in common desktop 3D printers. It operates by extruding filaments through a heated printhead. These filaments solidify upon cooling when they deposit on the substrate. Due to the behavior of the material required for FDM, only thermoplastic polymers can be used for this method.

Direct ink writing is another method that works by extrusion of visco-elastic materials that have shear thinning properties [59]. Cain et al. [60], for example, conducted 3D printing of MXenes using interfacial assembly of nanosheets made of Ti_3_C_2_T*_x_* with n-butylamine at the oil-water interface. MXene inks were negatively charged and extruded with a controlled flowrate into a viscous silicon oil-ligand matrix. When the mixing was finished, the oil-water interface was concentrated with ink using a structure similar to a 3D filament. Solidification is assisted by the evaporative ability of water, increasing the speed of printing.

**Figure 10:**
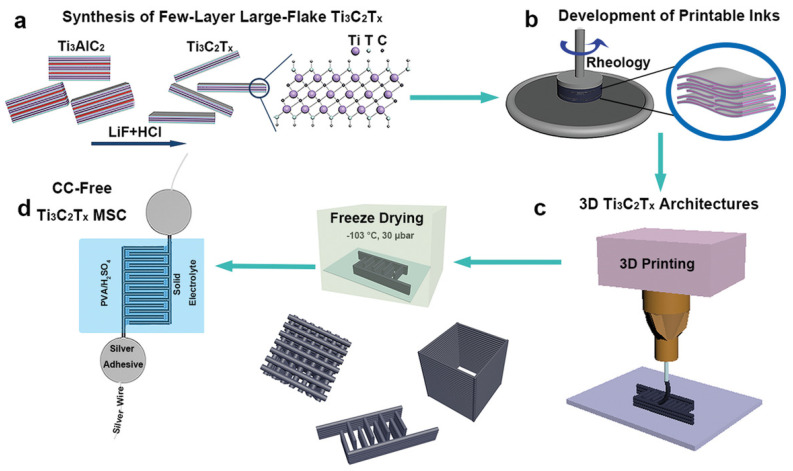
Schematic illustration of the manufacturing steps for 3D printing of freestanding MXene and MSC. (**a**) Synthesis process of Ti_3_C_2_T_x_ MXene. (**b**) Preparation of Ti_3_C_2_T_x_ inks at neutral pH. (**c**) The 3D printing of inks with ideal viscoelastic properties to manufacture various 3D design objects. (**d**) Solid-state MSC manufactured by 3D printing [61]. Copyright 2019, Wiley-VCH.

Wenji et al. [61] have formulated 2D Ti_3_C_2_T_x_ inks with suitable viscoelastic properties for 3D printing use. Figure 10 describes the manufacturing approach established for 3D printing of freestanding MXene structures and MSC. In addition to other advantages, 3D printing offers good thickness and geometry control, great flexibility of printing, economical feasibility, and eco-friendliness. Its primary drawback is the limited options when it comes to printable materials.

In addition to the four common printing techniques, there are other promising printing methods including flexographic printing, gravure printing, and spin coating, all of which are considered alternative candidates for the manufacturing of printable devices. However, these techniques have shown limited applications in the manufacturing of wearable electronic devices compared to the previously discussed printing methods. Table 1 summarizes some examples of 2D-materials inks and their printing methods.

## 6. Conclusions and Outlook

In this work, we have reviewed recent progress on 2D MXene inks, including synthesis procedures, inks formulation, and properties to printing methods for wearable devices, micro supercapacitor (MSC) electrodes, thin-film battery, and sensors. Screen printing, inkjet printing, stamping, and 3D printing are the primary methods for fabricating and developing wearable electronics with MXenes inks. The printing of these functional 2D materials offers more choices for preparing highly flexible and lightweight devices. Indeed, this technology is currently experiencing exponential growth.

Many experiments have explored standard printing methods and have enabled the fine tuning of several processes. Currently, the primary need of the MXene industry is economically viable processes that can develop flexible electronic devices on large scales and in bulk quantities to cater to the consumer electronic industry. MXenes have desirable optical, mechanical, electrochemical, and chemical properties.

A few issues remain poorly understood, primarily due to the fact that MXenes is a new field with only limited research conducted to date. In terms of formulation, there is still a vast challenge in converting 2D Mxene materials into printable inks. In addition, it is still hard to propose a common formulation for 2D Mxene inks with controllable physical and chemical properties that are in agreement with different requirements of existing printing technologies. Additional research should also be conducted on some of the critical parameters of MXenes, including the specific surface area of the substrates, viscosity, and volatility of different inks.

The stability of MXenes is yet another challenge potentially hindering MXenes from becoming industrially feasible. The inks have a small shelf life and require crucially controlled storage parameters. Moreover, the primary etching agent for the production of MXenes is hydrofluoric acid, which is highly toxic and corrosive. Due to these challenges, additional studies are needed to look into future developments of functional MXene materials and novel preparation methods for manufacturing more promising devices and applications.

## Figures and Tables

**Table 1:** Summary of the some reported 2D materials inks for numerous printing methods.

2D Materials	Solvents	Concentration (mg/mL)	Viscosity (mPa s)	Fabrication Method	Refs.
Ti_3_C_2_T_x_	water	22	1370	stamping	[56]
Ti_3_C_2_	water	30	10,000	direct writing	[44]
Ti_3_C_2_T_x_	ethanol	0.7	2	inkjet printing	[31]
Ti_3_C_2_T_x_	water	36	71	3D printing	[31]
Ti_3_C_2_T_x_/S	water	20	12,400	direct writing	[56]
Ti_3_C_2_T_x_	water	25	1000	blade coating	[62]
MXenes/SWCNTs	NMP	12	30,000	3D printing	[63]
MoS_2_	terpineol	0.1	40	inkjet printing	[64]
MoS_2_	ethanol/water	0.056	-	inkjet printing	[65]
MoS_2_	water	3–8	1.37	inkjet printing	[66]
BP	IPA and 2-butanol	5	2.2	inkjet printing	[10]
LFP/GO/LTO/GO	water	85	3 × 10^6^	3D printing	[67]

## Data Availability

Not applicable.
